# Unveiling the Power of Anticancer Drug Screening: A Clinical Case Study Comparing the Effectiveness of Hollow Fiber Assay Microtube Array Membrane (MTAM-HFA) in Breast Cancer Patients

**DOI:** 10.3390/cancers15102764

**Published:** 2023-05-15

**Authors:** Shih-Hsin Tu, Wan-Ting Huang, Chee Ho Chew, Amanda Lin Chen, Shou-Tung Chen, Jin-Hua Chen, Yi-Chen Hsieh, Chien-Chung Chen

**Affiliations:** 1 Department of Surgery, School of Medicine, College of Medicine, Taipei Medical University, Taipei 110, Taiwan; drtu@h.tmu.edu.tw; 2 Department of Surgery, Taipei Medical University Hospital, Taipei 11052, Taiwan; 3 Graduate Institute of Biomedical Materials and Tissue Engineering, Taipei Medical University, Taipei 11052, Taiwan; sandyhuang@mtamtech.com (W.-T.H.); chchew88@gmail.com (C.H.C.); 4 Translational Autoinflammatory Disease Section (TADS), Laboratory of Clinical Immunology and Microbiology (LCIM), National Institutes of Allergy and Infectious Diseases (NIAID), National Institutes of Health (NIH), Bethesda, MD 20892, USA; amanda.chen@nih.gov; 5 Comprehensive Breast Cancer Center, Changhua Christian Hospital, Changhua 50094, Taiwan; 1886@cch.org.tw; 6 Department of Medical Research, Changhua Christian Hospital, Changhua 50094, Taiwan; 7 Graduate Institute of Data Science, College of Management, Taipei Medical University, Taipei 11052, Taiwan; jh_chen@tmu.edu.tw; 8 Ph.D. Program in Medical Neuroscience, Taipei Medical University, Taipei 250, Taiwan; ychsieh@tmu.edu.tw; 9 Ph.D. Program in Biotechnology Research and Development, College of Pharmacy, Taipei Medical University, Taipei 250, Taiwan

**Keywords:** breast cancer, Microtube Array Membrane Hollow Fiber Assay (MTAM-HFA), personalized medicine, anti-cancer drug screening

## Abstract

**Simple Summary:**

The research paper reported on a newly developed screening technique called Microtube Array Membrane Hollow Fiber Assay (MTAM-HFA) used for determining the most effective medication for breast cancer patients. The study found that the MTAM-HFA approach had a high level of accuracy in predicting the patient’s response to the medication therapy, and there was a significant correlation between the screening outcome and the clinical outcome. The findings suggest that the MTAM-HFA technique has great potential for developing customized therapy for cancer care. With its ability to identify precise therapies for breast cancer patients, the MTAM-HFA screening method could transform cancer therapy, resulting in better outcomes for patients. The study provides valuable information for healthcare professionals and researchers working on breast cancer therapy research.

**Abstract:**

Breast cancer is a severe public health problem, and early treatment with powerful anticancer drugs is critical for success. The researchers investigated the clinical results of a novel screening tool termed Microtube Array Membrane Hollow Fiber Assay (MTAM-HFA) in breast cancer patients in this clinical investigation. In all trial participants, the MTAM-HFA was utilized to identify active medicines for the treatment of breast cancer. The MTAM-HFA was shown to be extremely useful in predicting patient response to anticancer medication therapy in this study. Furthermore, the substantial association between the MTAM-HFA screening outcome and the clinical outcome of the respective patients emphasizes the promise of this unique screening technology in discovering effective anticancer medication combinations for the treatment of breast cancer. These findings indicate that the MTAM-HFA has clinical significance and might be a valuable tool in the development of tailored therapy for cancer care. This study provides helpful information for physicians and scientists working on breast cancer therapy research. The potential benefits of employing MTAM-HFA to find accurate therapies for breast cancer patients might lead to enhanced personalized medicine approaches to cancer care, resulting in better patient outcomes. Overall, the MTAM-HFA screening approach has the potential to revolutionize customized cancer therapy, providing hope to both patients and physicians.

## 1. Introduction

Cancer is the biggest cause of mortality in the contemporary period worldwide. According to the International Agency for Research on Cancer (IARC), there were 17 million new cases and 9.5 million cancer deaths in 2018, with a projected 26.5 million new cases each year in 2040 [1,2]. Breast cancer was found to be the top cause of mortality among women and the sixth highest cause of death among cancers on a worldwide scale. Breast cancer cases increased by 33% between 2005 and 2015, with the following contributing variables accounting for 12.6% (population growth), 16.4% (aging population), and 4.1% (age-related cases) [3,4]. Current 5-year survival statistics vary according to the stage of breast cancer at diagnosis, with 99% survival for localized disease, 86% survival for regional illness, and 27% survival for metastatic disease [5,6].

Breast cancer (BC) is the most common cancer form in women worldwide. In fact, it is the most frequent cancer among women worldwide. This disease is exceedingly diverse, with four molecular subtypes, luminal A, luminal B, HER2-positive, and triple-negative breast cancer (TNBC) [7,8,9]. Current cancer therapy approaches include targeting these receptors with tailored endocrine and anti-HER2 treatments. Chemotherapy and radiation are frequently used in combination with these treatments, although they can have a negative impact on the patient’s quality of life [10,11,12,13]. Furthermore, these medicines may develop resistance in patients, rendering them useless. TNBC therapy is extremely difficult because of the lack of established treatments [14]. TNBC is distinguished by the lack of ER, PR, and HER2, making conventional BC therapy difficult to be utilized in the TNBCs [15,16]. As a result, researchers are working hard to create novel TNBC treatment alternatives, including immune-based treatments, targeted therapies, and combinations of chemotherapy and targeted therapies.

Breast cancer is still a major problem, especially in cases of triple-negative breast cancer (TNBC), when therapeutic choices are restricted. Current medicines can have serious negative effects, and patients might become resistant to them. As a result, there is an urgent need for more effective and customized TNBC therapies. Genetic profiling has emerged as a viable tool for guiding therapy options and improving TNBC patient outcomes [17]. Clinicians can better comprehend cancers’ distinctive traits and find new targets for therapy by studying their genetic makeup. As a result, genetic approaches to breast cancer management have been a focus of a significant investigation, particularly in cases of TNBC, where conventional therapeutic choices are ineffective [18,19,20].

Examples of such commercially available genetic assays are MamaPrint (Agendia, Inc., Irvine, CA, USA) and Genomic Grade Index (GGI; Affymetrix, Santa Clara, CA, USA), which use microarray technology to predict distant metastasis-free survival and treatment response in histological grade 2 patients [21,22]. Meanwhile, RT-PCR-based diagnostics, such as Oncotype DX (Genomic Health, Redwood City, CA, USA), can predict chemotherapy response in node-negative and ER+ breast cancer patients [23,24].

Despite the potential benefits, there are significant drawbacks to using genetic-based techniques to treat breast cancer patients. One significant disadvantage is the high expense, with these tests costing between $3000 and $4000. Because of the high cost, clinical usage of this technology is limited to individuals who can afford out-of-pocket expenditures [25,26,27]. Traditional pathology diagnoses, such as immunohistochemistry (IHC), are commonly employed in clinical settings but have drawbacks. Pre-analytical factors, such as fixation time, processing method, antigen type and concentration, and post-analytical elements, such as slide scoring systems and cutoffs for positive and negative findings, as well as post-analytical elements, such as cutoffs for positive and negative findings, can potentially affect the accuracy of the correct staging, classification, and treatment of breast cancer patients [28,29,30]. Furthermore, the evolution of innovative medicines now under research shows another major difficulty with IHC, genetic or biomarkers, which take a long time to uncover, validate, and deploy therapeutically. This gap between the availability of innovative treatments and the related prognostic prediction tests possibly delays the customized implementation of these novel therapies to breast cancer patients. As a result, it is critical to continue to investigate innovative techniques to improve the accuracy, accessibility, and cost of genetic approaches in order to enable their application in clinical practice and, ultimately, benefit patients.

In view of this situation, we developed the novel MTAM-based HFA (MTAM-HFA) that is capable of rapidly screening for the most suitable anti-cancer treatment plan for a particular patient in a personalized and timely manner [31]. Developed by the US National Cancer Institute (NCI) and combined with the novel new class of hollow fibers known as MTAM, the combined solution, MTAM-HFA, is capable of completing an entire test cycle within a clinically practical time frame of 14 days [31,32,33,34,35]. Interestingly, the unprecedented flexibility of MTAM-HFA makes it applicable in a wide range of cancer types and drug types, including immunotherapy, such as anti-PD1/anti-PDL1, while maintaining a very low and practical cost [32]. In this study, we are pleased to report the correlation between the screening outcome of the MTAM-HFA and the clinical outcome of breast cancer patients. This report summarizes the evidence and the corresponding clinical outcome between them.

## 2. Materials and Methods

### 2.1. Patient Inclusion Criteria

This clinical investigation was carried out in accordance with the ethical norms and regulations established by Taipei Medical University’s Institutional Review Board (IRB). (TMU-JIRB N201604012). The Taipei Medical University Hospital in Taipei City, Taiwan, was used for patient recruitment. This study’s inclusion criterion required female volunteers between the ages of 20 and 80 who had no prior history of breast cancer diagnosis and/or are currently pregnant. This study’s results were valid and accurate since these inclusion criteria were strictly followed. Furthermore, before participating in the clinical research, all patients were informed of the nature of this study and submitted written informed consent.

### 2.2. Study Design

**Figure 1:**
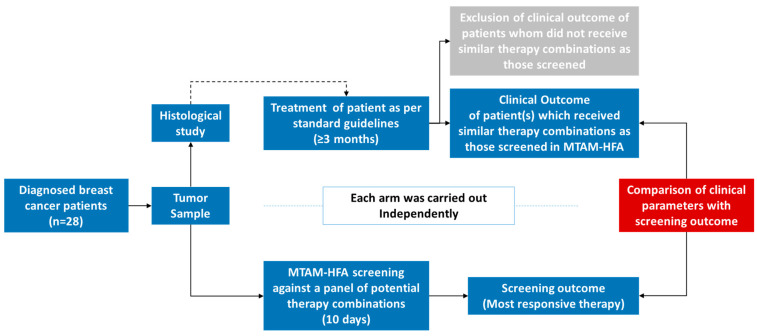
Overview of the structure of this study. Briefly, primary tumor samples derived from diagnosed breast cancer patients were divided equally for the following: (a) histological studies for staging, markers and etc., in line with the standard guidelines; and (b) for use in the MTAM-HFA anticancer drug screening where a panel of potentially used drugs was screened accordingly. After the completion of the treatment cycle on the patients’ end, the patients who received similar therapy combinations as those screened in the MTAM-HFA arm were compared against the respective outcome of the MTAM-HFA.

This study was based on an independent double-blind cohort clinical trial with the anti-cancer drug screening test (MTAM-HFA) and the treatment of breast cancer patients in accordance with National Comprehensive Cancer Network (NCCN) recommendations. The overview of this proposed study can be seen in Figure 1.

### 2.3. Acquisition of the Primary Breast Cancer Tissue

The fine needle aspiration (FNA) procedure was used to collect primary tissue samples. Specifically, 3–5 strips of primary breast cancer tissue (PBCT) measuring 2 cm in length were taken. The PBCTs were immediately placed in sterile Roswell Park Memorial Institute Medium (RPMI; Gibco, Taipei, Taiwan). The samples were subsequently sent to our laboratory at Taipei Medical University (Taipei City, Taipei, Taiwan) and kept at 4 degrees Celsius. When the PBCT samples arrived at the laboratory, they were promptly held at a constant temperature of 4 degrees Celsius for further examination. The MTAM-HFA anticancer drug screening was performed no later than 24 h after the PBCT samples arrived. The analysis was carried out in accordance with a well-established procedure, as described in [31,32], to evaluate the efficiency of several anticancer medicines against PBCT samples. To assure the accuracy and repeatability of the data, the MTAM-HFA test was performed in a controlled laboratory setting utilizing appropriate safety measures and quality control protocols.

### 2.4. Microtube Array Membrane-Hollow Fiber Assay (MTAM-HFA)

#### 2.4.1. Primary Breast Cancer Tissue (PBCT) Tissue Preparation

The procedure of the MTAM-HFA process was as described in previous works [31,36]. Briefly, the respective PBCTs were digested in a freshly prepared RPMI medium (Gibco, Taipei, Taiwan) containing 1% penicillin/streptomycin (P/S; Gibco, Grand Island, NY, USA). Next, the PBCTs were minced for a good 2 min until they resembled ‘mildly minced meat’. The minced PBCTs were then transferred into a freshly prepared RPMI medium (without PBS and P/S; Gibco, Grand Island, NY, USA), which contained collagenase (1000 unit/mL; Sigma, Burlington, MA, USA), Deoxyribonuclease I from bovine pancreas (0.1 mg/mL; Sigma, Burlington, MA, USA) and Hyaluronidase (300 units/mL; Sigma, Burlington, MA, USA). The entire PBCT mixture was then incubated at 37 degrees Celsius for 120 min. This was followed by the separation of the PBCTs from the medium with a sterile cell strainer (Clearance: 70 µm; Fisherbrand, MA, USA). The recovered PBCTs were then centrifuged (Thermo, Waltham, MA, USA) at 300 G for 5 min under ambient conditions, and the cell count was determined under an optical microscope (Zeiss, Dublin, CA, USA) with the assistance of Trypan Blue stain (Sigma, Burlington, MA, USA) and cell counter, hemocytometer (Fuxio Zentai, Taipei, Taiwan). Only PBCT samples with cell densities exceeding 1 × 10^5^ will now be taken into consideration for animal implantation. Below-target cell density PBCT samples will not be taken into consideration for further testing and will be destroyed in accordance with IRB policy. The resulting pellets were resuspended in freshly prepared RPMI medium (Gibco, Grand Island, NY, USA), achieving the desired cell density of no less than 1 × 10^5^ per 10 µL.

#### 2.4.2. Microtube Array Membrane-Hollow Fiber Assay Screening

**Figure 2:**
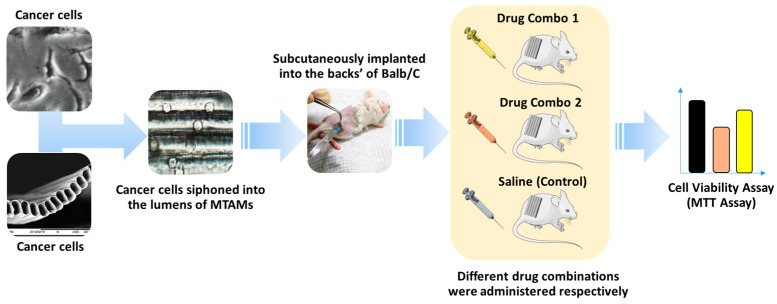
Illustration of the MTAM-HFA protocol, as described in [31,32]. Briefly, primary breast cancer samples were digested and siphoned into the MTAMs via capillary actions, with the respective ends heat-sealed. The cell-loaded MTAMs were then subcutaneously implanted into the backs of standard Balb/C mice. The respective drug combinations were administered accordingly, and after the completion of the treatment administration, MTT assay was carried out to determine the viability of each tumor sample in relation to the treatment received. A standard test cycle requires a short test duration of 10 days.

UV sterilized PSF MTAMs with dimensions of 0.5 cm × 1.5 cm were prepared, and PBCTs cell suspension in 10 µL was siphoned into the respective membranes. The ends of the PSF MTAMs were impulse-sealed, immediately transferred into sterile PBS, and swirled around to remove any excessive cells on the outer surface. Next, these cleaned PSF MTAMs with PBCTs encapsulated within were maintained in freshly prepared RPMI medium at 37 degrees Celsius under 5% CO_2_ atmospheric conditions and followed by implantation into the backs of the respective Balb/C mouse (Figure 2).

At the predetermined time points, the corresponding treatment (Doxorubicin at 3.75 mg/kg (A); Epirubicin at 6 mg/kg (E); Cyclophosphamide at 37.5 mg/kg (C); and Paclitaxel at 13 mg/kg (T)) were administered into study groups. The respective drugs were administered as follows: (1) AC (Q3D × 2) i.v., T (Q3D × 2) i.v.; (2) EC (Q3D × 2) i.v., T (Q3D × 2) i.v.); with the entire test cycle completed within 14 days. At the end of the test cycle, the respective PSF MTAMs were retrieved and subjected to MTT assay to determine the viability of the PBCTs encapsulated within. *t*-test statistical method was performed to compare the means of two groups in our study.

### 2.5. Response Evaluation Criteria in Solid Tumors (RECIST)

In this study, we compared the RECIST-based screening results of MTAM-HFA with the clinical outcomes of breast cancer patients. The clinical result was assessed utilizing CT scans taken before and after therapy to measure the tumor size change in primary breast cancer tissue (PBCT) samples, together with biomarker analyses. Based on established thresholds for changes in tumor size, the RECIST criteria were employed to identify tumor response and progression. In order to evaluate the effectiveness of various anticancer medications against PBCT samples, we largely evaluated the results of the cell viability test (MTT) in MTAM-HFA. We used RECIST criteria to evaluate the tumor response in the PBCT samples so that we could compare the screening result with the clinical result.

## 3. Results

**Figure 3:**
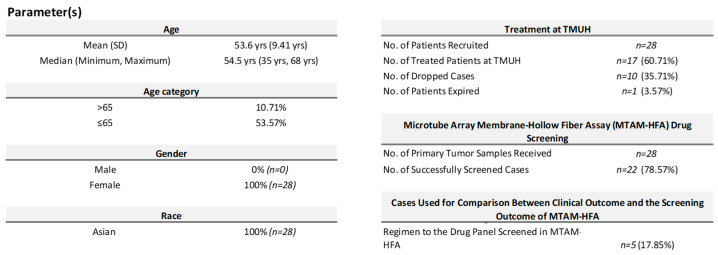
Demographic of the patients recruited in this clinical trial and the number of cases successfully screened with the MTAM-HFA anti-cancer drug screening solution for personalized medicine.

The enrollment and results of a clinical study that included a total of 28 participants are described in the aforementioned sentence. The patients who participated in the experiment had an average age of 53.6 ± 9.41 years. A total of 18 of the 28 patients who had therapy in line with National Comprehensive Cancer Network (NCCN) standards successfully completed their individual treatment regimens. Sadly, one participant passed away during the course of this study. The remaining 10 patients made the decision to discontinue their participation in this study, either by choosing to receive treatment elsewhere or as a result of a lack of clinical follow-up. It is significant to highlight that a number of elements, including individual circumstances or medical problems, may have had an impact on the decision to discontinue the clinical research by the respective individual participants (Figure 3).

**Figure 4:**
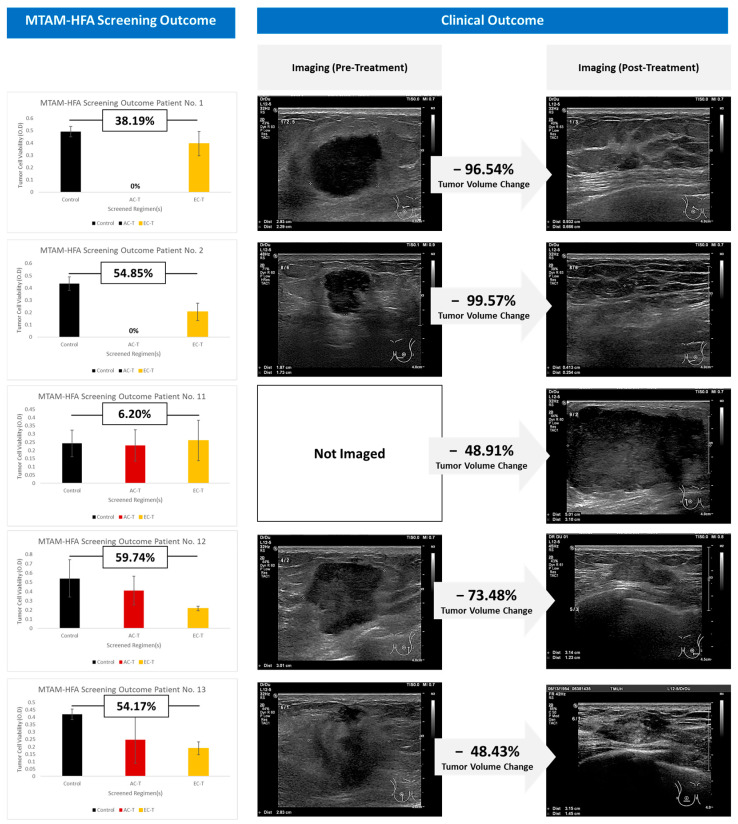
Illustration depicting the screening outcome of MTAM-HFA (**left column**), with its corresponding tumor volume change when compared against control. Clinical outcome, which depicts the imaging of the tumor before and after treatment, and its corresponding change in tumor volume, is depicted in percentage (**right column**).

Out of a total of 17, we found five patients in this research that had comparable treatment plans to those tested in the MTAM-HFA assay (Figure 4). Within the MTAM-HFA screening, the Doxorubicin–Cyclophosphamide–Paclitaxel (AC-T) and Epirubicin–Cyclophosphamide–Paclitaxel (EC-T) treatment regimens were compared to an untreated control group (saline injection) using the MTAM-HFA screening test.

According to the MTAM-HFA findings, when given the AC-T regimen, patients 1 and 2 had 100% less tumor viability than when given the EC-T regimen, which had viability reductions of only 38.19% and 54.85%, respectively. Patient number 11 saw a minimal decrease in tumor volume by 5.35% after receiving the AC-T regimen, whereas the EC-T treatment group experienced a 6.20% rise in tumor volume. Based on the results of the MTAM-HFA test, the EC-T treatment regimen was suggested for the final two instances, patients number 12 and 13. When compared to the control group, the tumor volume changes for these two individuals were 59.74% and 54.17%, respectively.

**Figure 5:**
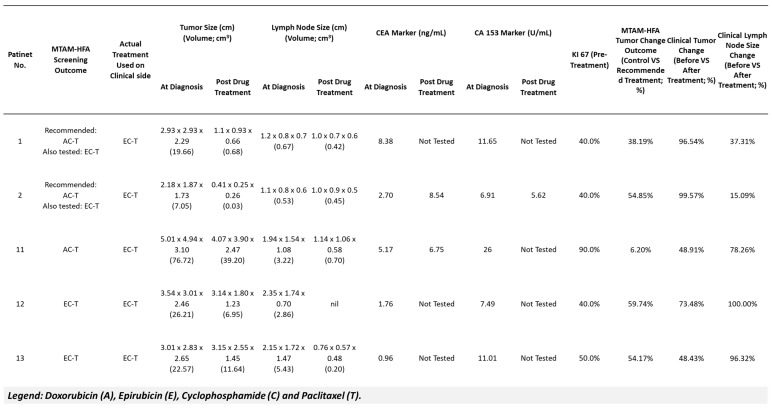
Overview of patients with similar screened drug regimens versus those that were clinically administered. For patients 1, 2, and 11, although the recommended treatment regimen of the MTAM-HFA is AC-T, part of the screened panel drugs is the EC-T regimen, and hence, the change in tumor of the EC-T regimen was used for the downstream analysis.

At the clinical end, all 5 patients received therapy with the EC-T regimen (Figure 4), in accordance with the NCCN recommendations and after consulting with their individual primary oncologists. The primary oncologist assessed changes in tumor volume using imaging and/or direct measurement. Significant tumor volume reductions of 96.54% and 99.57% were seen in patients 1 and 2, respectively. Both patients 12 and 13 responded well to the therapy, with tumor volumes decreasing by 73.48% and 48.43%, respectively. Following treatment, the tumor volume in Patient 11 decreased significantly by 48.91% from its initial value of 76.72 cm^3^ to 39.20 cm^3^ (Figure 5). However, because of her medical condition, a skilled oncologist only checked her tumor at the time of diagnosis rather than having it scanned.

In addition to tracking changes in tumor volume, we also assessed ancillary factors, including lymph node size (as shown in Figure 5). With a value of 100%, the data showed that patient 12 had the biggest change in lymph node volume. Patient 11 displayed a lymph node volume change of 78.26%, while patient 13 closely followed with a measurement of 96.32%. Readings for patients 1 and 2 were 37.31% and 15.09%, respectively. Additionally, we evaluated cancer markers, such as Carcinoembryonic Antigen (CEA) and Carbohydrate Antigen 15-3 (CA 153), where clinically appropriate. These indicators are crucial for tracking the development of breast cancer treatment.

## 4. Discussion

The high prevalence and death rate of breast cancer make it a serious global health problem. Chemotherapy is frequently used to treat breast cancer, but it can have substantial side effects, so it’s crucial to choose the best course of action for each patient. In this study, two commonly used chemotherapy regimens—doxorubicin–cyclophosphamide-paclitaxel (AC-T) and epirubicin–cyclophosphamide–paclitaxel (EC-T)—in patients with breast cancer were compared in this research using the MTAM-HFA screening assay.

For patients 1, 2, and 11 in the MTAM-HFA, our results showed that the chemotherapy regimen based on doxorubicin (AC-T) was more successful than the chemotherapy regimen based on epirubicin (EC-T) (Figure 5). With regard to this, we pinpointed five patients who had comparable treatment plans to those evaluated in the MTAM-HFA assay. When given the AC-T regimen, tumor cell response of patients 1 and 2 saw a 100% decrease in tumor volume, compared to 38.19% and 54.85% for the EC-T regimen, respectively. For the AC-T regimen, patient number 11 demonstrated a tumor volume reduction of 5.35 percent and a tumor volume growth of 6.20 percent when the EC-T regimen was administered. Both tumor samples of patient numbers 12 and 13 responded well to the EC-T treatment, with tumor volumes decreasing by 59.74% and 54.17%, respectively.

As this study is designed to be independent between the clinical end and the screening of MTAM-HFA, the respective primary oncologists opted to utilize the EC-T chemotherapy regimen in view of the clinical picture of the respective patients. In particular, doxorubicin is known to have a greater prevalence of hazardous side effects; specifically, Doxorubicin usage raises serious concerns about cardiotoxicity, with some studies indicating that up to 20% of individuals might have it after therapy [37]. This can result in a number of cardiac issues, such as myocardial infarction, arrhythmias, and congestive heart failure [38,39,40]. A severe adverse effect of doxorubicin therapy can also include myelosuppression or the inhibition of bone marrow activity, which raises the risk of infections and bleeding [41].

Similar anti-tumor efficacy to that of doxorubicin has been discovered for the closely related anthracycline epirubicin, but with less cardiotoxicity and myelosuppression [42,43]. Epirubicin is a safer alternative for some patients, especially those with pre-existing cardiac issues or who are more likely to develop cardiotoxicity, according to studies showing a reduced risk of cardiotoxicity linked with it than with doxorubicin [44,45,46]. Epirubicin is also a more palatable alternative for some individuals than doxorubicin since it has been demonstrated to cause less severe myelosuppression.

Tumor samples of patients 1, 2, and 11 (Figure 4) revealed that the doxorubicin chemotherapy regimen (AC-T) appears to be more efficient than epirubicin chemotherapy in MTAM-HFA screening. It is crucial to remember that the clinical result of patients who received the chemotherapeutic drug epirubicin (EC-T) resembles that of the drug doxorubicin in the MTAM-HFA screening. This could be a result of these anthracyclines’ shared modes of action, which include DNA intercalation and suppression of the topoisomerase II activity [47,48,49]. The intricacy of the human body and tumor microenvironment may not be adequately reflected by the MTAM-HFA screening because it was carried out in an in vivo model. Furthermore, compared to the in vivo model utilized in the MTAM-HFA screening, it’s likely that the patients who received epirubicin had traits or underlying problems that rendered them more receptive to the medication. The immune system of the patient, the existence of additional medical disorders, the stage and kind of cancer being treated, and other variables may also have an impact on the clinical result in patients. It is also critical to remember that, despite doxorubicin’s potential advantage over epirubicin in the MTAM-HFA screening, the drug is also more likely to cause hazardous side effects. In the end, the selection of a chemotherapy regimen should be based on the patient’s unique clinical circumstances as well as the possible advantages and disadvantages of each available treatment option.

Given that lymph node metastasis is a frequent occurrence in many forms of cancer, the lymph node size change is a crucial metric to assess the effectiveness of chemotherapy [50,51]. In this study, we discovered that the lymph node size changes in the patients who received the EC-T regimen varied in severity, with patient 1 showing a decrease of 37.31%, patient 2 showing a slight decrease of 15.09%, patient 11 showing an increase of 78.26%, patient 12 showing a complete reduction of 100%, and patient 13 showing a reduction of 96.32%. The clinical lymph node size changes in patients 1 and 2 were consistent with the tumor volume change seen in MTAM-HFA screening. Patients 11, 12, and 13, on the other hand, had a decrease in tumor volume but different changes in lymph node size, with patients 12 and 13 exhibiting entire and almost complete decreases in lymph node size, respectively.

This hypothesis is supported by the data from this investigation, which shows that changes in tumor volume and lymph node volume in clinical patients corresponded with the results from the MTAM-HFA model in terms of the response of cancer tumors and/or lymph node size toward a particular chemo regimen. Patients who showed the greatest tumor volume change in the MTAM-HFA model also demonstrated the greatest tumor volume change in the clinical context, indicating that the MTAM-HFA model may be beneficial in predicting treatment results. Despite its potential benefits, the MTAM-HFA technique has some drawbacks to consider. This technique necessitates a minimum cell population of 1 × 10^4^, which may not always be possible, especially when tumor samples are small or sparse, which makes this technique more applicable in later stages of cancer.

Overall, the information reported in this study emphasizes the MTAM-HFA model’s promise for drug screening and prognosticating treatment results. This model can be used to identify promising drug candidates and provide a more personalized approach to cancer treatment.

## 5. Conclusions

Overall, this study demonstrates that MTAM-HFA may be a useful tool for anticipating cancer patients’ treatment reactions. The outcomes of the MTAM-HFA screening were significantly correlated with the tumor and lymph node volume changes seen in clinical patients, according to our findings. The general pattern of medication response prediction remained the same across the screening and clinical outcomes, even if the precise proportion of volume changes may not have matched exactly. These results demonstrate the potential of MTAM-HFA in personalized medicine and may pave the way for the development of more precise and efficient cancer therapies. However, because this study was double-blind and cohort-based, the sample size was constrained, and only patients with comparable chemo regimens were included for comparison. Therefore, to fully demonstrate the capability of the MTAM-HFA to predict medication response and, perhaps, guide customized treatment, future clinical research should entail larger-scale intervention clinical trials.

## Data Availability

The data presented in this study are available in this article.
